# Supplementary material for: DNA damage and antioxidant capacity in COPD patients with and without lung cancer
Source: PLoS One. 2022 Nov 3;17(11):e0275873. doi: 10.1371/journal.pone.0275873 (PMC9632772; doi:10.1371/journal.pone.0275873)
Supplement: Attachment — Submitted filename: Response to Reviewers.pdf [file pone.0275873.s001.pdf]

Dear Editor Emre Avci,

We appreciate your letter and reviewer' comments concerning our manuscript entitled: **"DNA damage and antioxidant capacity in COPD patients with and without lung cancer**". Those comments are all valuable and very helpful for revising and improving our paper. Based on your comment and request, we have made modification in the original manuscript. We attached the revised manuscript in a separate file entitled 'Revised Manuscript with Track Changes' and we uploaded a separate file entitled 'Manuscript' without tracked changes. Please see below the answers to reviewer#2 and the acknowledgments for reviewers #1 and #3. We hope that our manuscript is now acceptable for publication in this renowned leading journal.

Best regards,

Camila Santos

Journal Requirements:

1. Please ensure that your manuscript meets PLOS ONE's style requirements, including those for file naming. The PLOS ONE style templates can be found at https://journals.plos.org/plosone/s/file?id=wjVg/PLOSOne_formatting_sample_main_body.pdf and https://journals.plos.org/plosone/s/file?id=ba62/PLOSOne_formatting_sample_title_authors_affiliations.pdf

R: We made modifications to the manuscript to suit PLOS ONE's style requirements.

2. In your Data Availability statement, you have not specified where the minimal data set underlying the results described in your manuscript can be found. PLOS defines a study's minimal data set as the underlying data used to reach the conclusions drawn in the manuscript and any additional data required to replicate the reported study findings in their entirety. All PLOS journals require that the minimal data set be made fully available. For more information about our data policy, please see http://journals.plos.org/plosone/s/data-availability
Upon re-submitting your revised manuscript, please upload your study's minimal underlying data set as either Supporting Information files or to a stable, public repository and include the relevant URLs, DOIs, or accession numbers within

your revised cover letter. For a list of acceptable repositories, please see http://journals.plos.org/plosone/s/data-availability#loc-recommended-repositories

Any potentially identifying patient information must be fully anonymized.

Important: If there are ethical or legal restrictions to sharing your data publicly, please explain these restrictions in detail. Please see our guidelines for more information on what we consider unacceptable restrictions to publicly sharing data: http://journals.plos.org/plosone/s/data-availability#loc-unacceptable-data-access-restrictions .

Note that it is not acceptable for the authors to be the sole named individuals responsible for ensuring data access.

We will update your Data Availability statement to reflect the information you provide in your cover letter.

R: The data cannot be shared due to potentially identifying or sensitive information. Future researchers will be able to request the data used in this study through the contact of the local ethics committee of São Paulo State University: Chácara Butignolli, s / n, Botucatu, São Paulo - Brazil. Zip code: 18618970. telephone: 3880-1609. e-mail: cep.fmb@unesp.br. Additionally, the study ethics approval may be requested through the conep portal (https://Plataformabrasil.saude.gov.br/).

3. Please review your reference list to ensure that it is complete and correct. If you have cited papers that have been retracted, please include the rationale for doing so in the manuscript text, or remove these references and replace them with relevant current references. Any changes to the reference list should be mentioned in the rebuttal letter that accompanies your revised manuscript. If you need to cite a retracted article, indicate the article's retracted status in the References list and also include a citation and full reference for the retraction notice.

R: The references used in the manuscript have been updated.

Reviewer's comments:

Reviewer #1: The manuscript by Freitas dos Santos et al. deals with COPD patients and their vulnerability to lung cancer due to increased DNA damage and altered antioxidant status. Obtained results may provide a contribution to cancer studies. The aim of the study is remarkable. Although findings are not sufficient to claim exact correlation between cancer and oxidative status of COPD patients, in my opinion, the scientific level of the manuscript is appropriate for PlosOne in its current form.

R: We would like to thank you for the opportunity to review our manuscript. We are flattered by the recognition of the importance of our manuscript. As mentioned, although the results were not enough to prove our initial hypothesis, we were able to bring this perspective as a contribution to the scientific community, which can culminate in new hypotheses and answers.

Reviewer #2: It is a valuable study which is investigating the DNA damage and oxidative stress and their relationship in patients with COPD with and without lung cancer. But I have some suggestions that I have stated below.

- Introduction: The importance of oxidative stress markers in cancer should be briefly mentioned.

R: We would like to thank you for highlighting that is a valuable study. We appreciate the reviewer's suggestions. We tried our best to clarify all the issues raised. We are confident that all the suggestions/recommendations and changes made (highlighted in the text) were very important to improve the manuscript.

In relation to your suggestion, we briefly mentioned it in the Introduction section (3rd paragraph), as requested.

Oxidative stress markers are identified as possible diagnostic predictors of cancer [1]. It has already been reported higher malondialdehyde (MDA) level, an important lipid peroxidation marker, in lung cancer patients than in controls [1]. Decreased antioxidant status suggest a link between oxidative stress and malignant transformation; it has been shown a relationship between MDA levels and more advanced clinical stages of the disease, which could be used for staging [2].

- Material and Methods: The selection criteria of the **control group** are not clear from the text; more detailed information should be given.

R: We appreciate the suggestion and we describe more details in the Materials and methods section (1st paragraph), as requested.

Individuals who were active smokers, without comorbidities and with spirometry tests results within the normal range were included in the control group.

- Why MDA and HAP have been as markers? Could you explain with references?

R: We appreciate the question and explain that oxidative stress occurs due to an imbalance between the levels of reactive nitrogen/oxygen species (RNOS) and antioxidants [1]. Please note that oxidative stress should be detected by both oxidized and antioxidative markers to have a reliable scenario. When the production of RNOS is not controlled, it leads to cellular lipid, protein, and DNA damage in biological systems. To measure this imbalance, we used MDA, which is a gold-standard marker of oxidative stress (related to lipid peroxidation) [3] and HAP, used as an antioxidant marker [4]. MDA is one of the most preferred and reliable markers for oxidative stress, derived from end-products of the peroxidative degradation of polyunsaturated fatty acids and it has been shown at high levels in different types of cancer, showing its usefulness as a possible tumor marker [1, 5]. In addition, MDA is easily detected in blood/plasma, and appears to be the most mutagenic product of lipid peroxidation [5, 6]. HAP measures the antioxidant capacity in the aqueous compartment of plasma to assess endogenous antioxidant defense, which clears free radical/RONS activity and accumulation in cells and maintain redox balance. [7]. To clarify, we briefly added information and references about both methods in the revised manuscript.

To evaluate oxidative stress, both MDA, a gold-standard marker of lipid peroxidation [3], and hydrophilic antioxidant performance (HAP) that measures the antioxidant capacity in the aqueous compartment of plasma to assess antioxidant defense, which clears free radical/RNOS activity and accumulation in cells and maintain redox balance [7], were evaluated as biomarkers.

A 100-µL aliquot of the plasma was used for the MDA concentration analysis in duplicate, according to a previous protocol [4], with slight modifications. Briefly, we add 700 µL of 1% orthophosphoric acid and 200 µL of thiobarbituric acid (42 mM) to the sample and then heated it for 60 min in a water bath at 100°C; the sample was cooled on ice immediately after. Two hundred microliters were transferred to a 2-mL tube containing 200 µL of NaOH-methanol (1:12 v/v). The sample was vortex-mixed for 10 s and centrifuged for 3 min at 13000 g. We transferred 200 µL of the supernatant to a 300-µL glass vial and injected 50 µL onto the column. Shimadzu LC-10D system (Shimadzu Corporation, Kyoto, Japan) was used for high-performance liquid chromatography, with a C18 Luna column (5 µm, 150 × 4.60 mm, Phenomenex Inc., Torrance, CA, USA), a Shimadzu RF-535 fluorescence detector (excitation: 525 nm, emission 551 nm), and 0.5 mL/min flow of KH2PO4 (1 mM, pH 6.8). MDA was quantified by the area determination of the peaks in the chromatograms relative to a standard curve of known concentrations [8].

- Results: In Table 1, too many spirometry results have been shared, although they are not mentioned in the text. Please simplify the data in table 1.

R: We agreed with the reviewer and excluded some data about the same variable from the table. The information is more succinct and does not change the interpretation of the results.

Table 1. General characteristics of patients in the study

**Variables**	**Control (n = 18)**	**COPD without cancer (n = 18)**	**COPD with cancer (n = 18)**	**p Value**
Gender (male/female)	11/7	13/5	12/6	0.779
Age (years)	53.3 ± 11.8[a]	65.0 ± 10.7[b]	70.2 ± 9.2[b]	**<0.001**
GOLD I/II/III (n)	-	5/8/5	4/7/7	0.572
Current smokers (n/%)	12/66	8/44	9/50	0.380
Smoking history (pack-years)	32.5 (17.0-60.0)[a]	49.0 (38.5-65.0)[ab]	69.0 (50.0-106.0)[b]	**0.014**
CO (ppm)	10.0 (0-18.0)	1.5 (0.0-8.0)	1.0 (0-3.0)	0.071
FVC (% predict)	90.8 ± 20.7	84.1 ± 20.9	75.8 ± 20.1	0.101
FEV_1 (% predict)	88.3 ± 21.2[a]	63.2 ± 20.6[b]	59.6 ± 20.2[b]	**<0.001**
FEV_1/FVC	0.80 ± 0.06 [a]	0.57 ± 0.08 [b]	0.59 ± 0.09 [b]	**<0.001**
BMI (kg/m^2)	27.5 ± 5.0[a]	22.0 ± 4.7[b]	21.7 ± 2.5[b]	**<0.001**
FFM (kg)	52.0 ± 9.8	44.2 ± 11.4	43.7 ± 6.4	0.062
MRC	0.5 ± 0.6[a]	1.4 ± 1.1[ab]	1.7 ± 1.3[b]	**0.011**
SpO_2 (%)	97.0 (95.0-98.0)[a]	95.0 (93.0-97.0)[b]	94.0 (92.0-96.0)[b]	**0.006**

Values expressed as X ± SD or median (quartile 1 - quartile 3). COPD I/II/III: mild/moderate/severe (GOLD: Global Initiative for Chronic Obstructive Lung Disease). FVC = forced vital capacity (% of predicted); FEV_1 = forced expiratory volume in the first second (% of predicted); BMI = body mass index; FFM = fat-free mass; ppm: parts per million; MRC = Modified Research Council; SpO_2 = pulse oximetry. a, b: different letters indicate statistically significant difference. $p < 0.05$. $\chi 2$, ANOVA or Kruskal-Wallis test.

- Are there associations between the number and duration of smoking and the results? It should be analyzed.

R: We agree with the reviewer, and we included the relationship between smoking history and the results. It was possible to relate the higher levels of DNA damage in the COPD with cancer group with greater exposure to cigarettes, given the greater number of pack-years found in this group, compared to the control group. In relation to the COPD-only group, there were no significant differences, as much in smoking history as DNA damage. We will include it in the Discussion section (2nd paragraph), as requested.

In our sample, there was no significant difference in smoking history between the COPD and COPD cancer groups, but there was a difference between the control and COPD cancer groups. Based on this information, it is possible to relate the higher levels of DNA damage in the COPD group with the greater exposure to cigarettes, given the greater number of pack-years, as shown in Table 1.

- Is there an association between the clinical progression and the results? It should be analyzed, if not possible please specified as a limitation.

R: We appreciate the suggestion, but it was not possible to follow the clinical evolution of the patients in the sample. Thus, we will include it as a limitation of the study in the Discussion section (8th paragraph), as requested.

Additionally, we were not able to follow the clinical progression of the patients in the samples, which limited the possible association with the results found.

- Discussion: Analyzes have been performed according to the information about age, gender etc. and shared in the tables by authors. But have not been mentioned in the discussion part. Please, mention briefly.

R: We agreed with the reviewer and included this information in the Discussion section (8th paragraph). The analysis in relation to the age and BMI of the individuals in the sample is pointed out as a limitation of the study. The mean age is higher in the COPD groups with and without lung cancer, which influences oxidative markers and DNA damage due to physiological changes with age. In addition, the controls showed a higher BMI than patients with COPD with or without cancer.

One limitation of this study is related to the average age of the control group, which was lower than that of the other groups. This fact may have culminated in differences in the levels of DNA damage and percentage of antioxidants from the natural process of aging, and not only in relation to the presence of COPD and the process of carcinogenesis

[9]. In addition, the controls showed a higher BMI than patients with COPD with or without cancer. This may have been associated with high levels of DNA damage in multiple organs [10] in patients in the control group, approximating the results between them and the patients with COPD without lung cancer. In 2017, Gariballa et al. suggested that the measurement of waist circumference is a stronger predictor than the BMI; thus, our study findings may not have shown the effects of obesity accurately [11]. The patients with COPD (eutrophic) with lung cancer presented significantly higher levels of DNA damage than the controls (overweight), suggesting that the weight seemed to have a minimal influence on DNA damage. Additionally, we were not able to follow the clinical progression of the patients in the samples, which limited the possible association with the results found.

- The discussion has been based on smoking by the authors. But, correlation analysis with smoking has not been performed. İmprove the discussion section according to analysis suggested in the results section.

R: We agreed with the reviewer and included the relationship between smoking history and results in the Discussion section (2nd paragraph), as requested.

In our sample, there was no significant difference in smoking history between the COPD and COPD cancer groups, but there was a difference between the control and COPD cancer groups. Based on this information, it is possible to relate the higher levels of DNA damage in the COPD group with the greater exposure to cigarettes, given the greater number of pack-years, as shown in Table 1.

- What are the authors' recommendations regarding the results obtained with limited patient data? Please improve the conclusion part at the end of the manuscript.

R: We agreed with the reviewer and included the recommendations based on our results and based on the analysis of the limitations of this study (Discussion section, 9th paragraph). Among them, we can mention new inclusion criteria for samples such as age, BMI, current smoker, and smoking history. In addition, we recommend monitoring the clinical evolution of each individual in order to relate their current clinical stage with new levels of DNA damage and oxidative stress markers.

Finally, based on our results, patients with COPD and lung cancer presented increased levels of DNA damage compared to the controls. The MDA indicated high levels of oxidative stress in individual with COPD and lung cancer, but there was no difference compared to the other groups. The percentage of HAP was lower in patients who had an association of lung cancer and COPD compared to controls and patients with

COPD without cancer. Further studies should be conducted to address the limitations of the present study. We recommend including selection criteria such as age, BMI, current smoker and, smoking history for better standardization for all individuals in the sample. We also recommend monitoring the clinical course of each individual in order to relate their current clinical stage to new levels of DNA damage and markers of oxidative stress.

Reviewer #3: The manuscript is well written. Authors have evaluated the DNA damage in COPD and COPD with cancer patients in comparison to control. The results were presented with appropriate statistical evaluation.

R: First, we would like to thank you for the opportunity to review our manuscript. We are grateful for the points mentioned. Our objective is to present clear and easy-to-interpret language. Our statistical evaluation was carefully addressed, in addition to sending tables containing the data used during the research, ensuring reproducibility.

References

1. Jelic MD, Mandic AD, Maricic SM, Srdjenovic BU. Oxidative stress and its role in cancer. J Cancer Res Ther. 2021;17(1):22-8. doi: 10.4103/jcrt.JCRT_862_16. PubMed PMID: 33723127.
2. Pande D, Negi R, Khanna S, Khanna R, Khanna HD. Vascular endothelial growth factor levels in relation to oxidative damage and antioxidant status in patients with breast cancer. J Breast Cancer. 2011;14(3):181-4. Epub 20110929. doi: 10.4048/jbc.2011.14.3.181. PubMed PMID: 22031798; PubMed Central PMCID: PMCPMC3200512.
3. Spirlandeli AL, Deminice R, Jordao AA. Plasma malondialdehyde as biomarker of lipid peroxidation: effects of acute exercise. Int J Sports Med. 2014;35(1):14-8. Epub 20130614. doi: 10.1055/s-0033-1345132. PubMed PMID: 23771832.
4. Braz MG, Braz LG, Freire CMM, Lucio LMC, Braz JRC, Tang G, et al. Isoflurane and Propofol Contribute to Increasing the Antioxidant Status of Patients During Minor Elective Surgery: A Randomized Clinical Study. Medicine (Baltimore). 2015;94(31):e1266. doi: 10.1097/md.0000000000001266. PubMed PMID: 26252290; PubMed Central PMCID: PMCPMC4616612.
5. Rahal A, Kumar A, Singh V, Yadav B, Tiwari R, Chakraborty S, et al. Oxidative stress, prooxidants, and antioxidants: the interplay. Biomed Res Int. 2014;2014:761264. Epub 20140123. doi: 10.1155/2014/761264. PubMed PMID: 24587990; PubMed Central PMCID: PMCPMC3920909.
6. Ayala A, Muñoz MF, Argüelles S. Lipid peroxidation: production, metabolism, and signaling mechanisms of malondialdehyde and 4-hydroxy-2-nonenal. Oxid Med Cell Longev. 2014;2014:360438. Epub 20140508. doi: 10.1155/2014/360438. PubMed PMID: 24999379; PubMed Central PMCID: PMCPMC4066722.
7. Yeum KJ, Russell RM, Krinsky NI, Aldini G. Biomarkers of antioxidant capacity in the hydrophilic and lipophilic compartments of human plasma. Arch Biochem Biophys. 2004;430(1):97-103. doi: 10.1016/j.abb.2004.03.006. PubMed PMID: 15325916.
8. Vasconcelos SML, Goulart MOF, Moura JBdF, Manfredini V, Benfato MdS, Kubota LT. Espécies reativas de oxigênio e de nitrogênio, antioxidantes e marcadores de dano oxidativo em sangue humano: principais métodos analíticos para sua determinação. Química Nova. 2007;30(5):1323-38. doi: 10.1590/S0100-40422007000500046.

9. Shimizu I, Yoshida Y, Suda M, Minamino T. DNA damage response and metabolic disease. Cell Metab. 2014;20(6):967-77. Epub 2014/11/13. doi: 10.1016/j.cmet.2014.10.008. PubMed PMID: 25456739.
10. Setayesh T, Nersesyan A, Mišík M, Ferk F, Langie S, Andrade VM, et al. Impact of obesity and overweight on DNA stability: Few facts and many hypotheses. Mutat Res. 2018;777:64-91. Epub 2018/07/11. doi: 10.1016/j.mrrev.2018.07.001. PubMed PMID: 30115431.
11. Gariballa S, Alkaabi J, Yasin J, Al Essa A. Oxidative damage and associated inflammatory risk factors in obese Emirati women. Body mass index versus waist circumference. Saudi Med J. 2017;38(9):960-4. doi: 10.15537/smj.2017.9.19629. PubMed PMID: 28889156; PubMed Central PMCID: PMCPMC5654032.
